# In-situ Interprofessional Perinatal Drills: The Impact of a Structured Debrief on Maximizing Training While Sensing Patient Safety Threats

**DOI:** 10.7759/cureus.4096

**Published:** 2019-02-19

**Authors:** Joy A Greer, Gayle Haischer-Rollo, Donald Delorey, Rebecca Kiser, Timothy Sayles, Jennifer Bailey, Colleen Blosser, Reginald Middlebrooks, Christopher S Ennen

**Affiliations:** 1 Obstetrics and Gynecology, Naval Medical Center, Portsmouth, USA; 2 Pediatrics, Brooke Army Medical Center, San Antonio, USA; 3 Psychology, Naval Medical Center, Portsmouth, USA; 4 Anesthesiology, Naval Medical Center, Portsmouth, USA

**Keywords:** simulation, multi-disciplinary

## Abstract

Introduction

In-situ interprofessional emergency team training improves participants’ with confidence and knowledge and identifies latent safety threats. This study examined the impact of a structured debrief on an interprofessional perinatal team’s ability to identify latent safety threats and assess competency in managing perinatal emergencies. It was hypothesized that latent safety threats would be reduced and checklist compliance would increase during subsequent in-situ perinatal team training.

Methods

Two in-situ training sessions were held six months apart. The perinatal emergency response team provided care for a standardized patient with preterm twin gestation. Each session included off-ward delivery and resuscitation of the first infant, transportation to appropriate inpatient units, cesarean delivery, and resuscitation of the second twin. Postpartum hemorrhage ensued, requiring massive transfusion protocol activation. Medical experts assessed team performance with critical action checklists. A structured debrief identified latent safety threats, developed action plans, and reviewed checklist compliance. Checklist compliance rates were analyzed using a z-ratio test.

Results

The first training session: seven teams (75 staff) completed 75% (292/391) critical action checklist items and identified 34 latent safety threats. Second training session: four teams (45 staff) completed 89% (94/106) critical action checklist items. Ten latent safety threats were mitigated during the second session. Utilizing a z-ratio, a significant difference was detected between the overall checklist compliance rates of the two sessions, z = -3.069, p = .002. Post-hoc power calculation was <10%.

Conclusions

In-situ interprofessional perinatal emergency team training is feasible, identifies latent patient safety threats, and may improve team competency.

## Introduction

Perinatal care accounts for 49% of inpatient care provided to female Military Health System (MHS) beneficiaries. In 2015, approximately one-third of the 43,000 deliveries in the MHS occurred at US Navy Military Treatment Facilities (MTFs) [1]. While obstetric emergencies are frequently unpredictable, improving team effectiveness significantly impacts clinical outcomes for both the mother and the infant [2]. Obstetric hemorrhage complicates 4%-6% of all deliveries and remains a leading cause of treatable maternal morbidity and mortality in the United States. It is also responsible for an average of one maternal death every four minutes worldwide [3].

Postpartum hemorrhage (PPH) is treatable and PPH safety programs and bundles have been associated with improved outcomes [4-5]. An integral component of these bundles includes healthcare simulation with enhanced patient scenarios and advanced task trainers targeted at improving perinatal team performance and decreasing PPH rates and, subsequently, maternal morbidity [6-8]. Institutional perinatal leadership raised concerns that latent safety threats may exist for an obstetric patient who delivers precipitously and then experiences postpartum hemorrhage prior to being admitted to the hospital and desired to test the current system navigation and processes [9-10].

Several studies report using in-situ simulation scenarios to identify latent safety threats within a unit or health system. These threats can be identified through the debriefing process [11-15]. Latent safety threats are “system-based threats to patient safety that can materialize at any time and are previously unrecognized by healthcare providers, unit directors, or hospital administration" [16]. Structured debriefing provides participants a unique learning opportunity to process the simulation experience, analyze thoughts, find meaning in the simulation, and better understand the connections between knowledge gained in simulation and real-life situations [17-19]. The lessons learned through the structured debriefing process can then be applied to inform patient care and hopefully improve patient outcomes. Currently, only limited studies report the use of structured debriefing during multi-disciplinary team training as a means to increase emergency checklist [20-21].

To address these concerns, the authors developed an in-situ interprofessional perinatal team simulation training scenario incorporating postpartum hemorrhage to assess system processes and team performance during a simulated perinatal emergency across the continuum of care. We hypothesized that in-situ interprofessional perinatal team simulation training would improve team function and performance. We further hypothesized that integrated formal structured debriefing would reveal unrecognized latent safety threats, identify gaps in our patient safety systems that could be addressed prior to impacting an actual patient, and increase emergency checklist compliance.

## Materials and methods

The study was determined to be Non-Human Subjects Research by the local institutional review board and was judged as exempt. The study also adhered to the framework described by the Standards for Quality Improvement Reporting Excellence (SQUIRE) guidelines [22].

Study design

The study utilized two, distinct, one-hour, in-situ interprofessional perinatal emergency simulation training scenarios, which were administered approximately six months apart in 2016. Members from the departments of Healthcare Simulation and Bioskills Training Center, Women’s Health, Anesthesia, Pediatrics, and Maternal Infant Nursing at Naval Medical Center Portsmouth developed and implemented two in-situ interprofessional perinatal emergency simulation scenarios to identify potential system latent safety threats in patients with obstetric hemorrhage. The objectives were to test team communication across disciplines, determine competency in responding to perinatal emergencies, and examine emergency checklist compliance between the two training sessions. The intervention of a structured debrief was administered following the completion of the first in situ perinatal emergency simulation training scenario.

Scenario for the two training sessions

The developed scenario required the perinatal emergency response team (consisting of providers and trainees from the departments of anesthesia, obstetrics, pediatrics, nursing, and ancillary services) to provide patient care for a simulated pregnant woman with a preterm twin pregnancy who delivered the first infant off the labor and delivery unit prior to hospital admission. The team needed to transport the mother with the undelivered second twin and the newborn infant to appropriate inpatient units and deliver the second twin by cesarean delivery. The mother then experienced a postpartum hemorrhage requiring the activation of the massive transfusion protocol for the mother and emergency release of blood for the second infant. If blood transfusion was not initiated within 15 minutes after requesting the massive transfusion from the blood bank, the mother progressed to cardiac arrest and recovered with appropriate Advanced Cardiac Life Support interventions, including blood product administration. Team competency was assessed using critical action checklists by trained proctors from obstetrics, anesthesia, pediatrics, and nursing. A separate training team for each session was identified from those assigned to provide clinical care on the units for the day to prevent the in-situ training from impacting ongoing clinical care. One of the three available labor and delivery operating rooms was blocked for the operative portion of the in-situ training.

The scenario started with a standardized patient using the MamaNatalie® birthing simulator (Laerdal, Wappingers Falls, New York, United States of America) in a remote location of the hospital. A perinatal emergency drill was announced overhead. Assigned team members of the perinatal emergency response team responded to care for the patient, a 24-year-old primigravida with a twin gestation at 28 weeks gestation who was feeling the urge to push. Upon the team’s arrival, the patient had preterm premature rupture of membranes followed by the delivery of the first twin. The pediatrics team used a newborn mannequin (SimNewB®, Laerdal, Wappingers Falls, New York, United States of America) for the initial resuscitation of the first twin. The standardized patient was then transferred to the labor and delivery unit, as the cervix remained completely dilated with a bulging bag but no palpable, presenting fetal part. Upon the standardized patient’s arrival on the labor and delivery unit and the performance of an ultrasound to discover fetal presentation (transverse, back-down), the scenario continued using a birthing mannequin (NOELLE® Maternal and Neonatal Birthing Simulator, Gaumard® Scientific, Miami, Florida, United States) modified with an insert to facilitate a cesarean delivery of the second twin and a second newborn mannequin (SimNewB®, Laerdal, Wappingers Falls, New York, United States of America) to enable the resuscitation. Obstetric and anesthesia care for the maternal patient continued until the blood products arrived and began to be transfused.

The scenario took approximately one hour to run and was followed by one hour of debrief (30 minutes within each specialty team separately: obstetrics, pediatrics, nursing, and anesthesia, as well as 30 minutes for a large group debrief to discuss team function according to TeamSTEPPS™ (Strategies and Tools to Enhance Performance and Patient Safety) principles [23]. In addition, training and system gaps that needed to be addressed were identified. Surveys were distributed to learners to assess the simulation exercise. Patient satisfaction surveys were compared six months prior to the training and five months after the identification of threats to evaluate patient perceptions of their care.

Statistical analysis

Descriptive statistics were used to determine checklist compliance rates, participant and patient satisfaction survey outcomes, latent safety threats, and participant confidence and patient satisfaction. A z-ratio test was used to determine if there was a significant change in the overall checklist compliance rates between the two training sessions. Mann-Whitney tests were used to analyze participant survey responses and an independent t-test was used to assess patient satisfaction scores. Finally, a post hoc power analysis was performed to determine the power of the study, the likelihood of the presence of a type I or type II error, and what sample size would be needed for future studies.

## Results

A total of 75 staff members (seven teams) participated in the initial training over seven sessions, including representation from obstetrics, pediatrics, nursing, anesthesia, and respiratory therapy, based on hospital instruction for staffing a perinatal emergency response team. One of the eight planned training sessions was canceled due to emergent patient care needs requiring the use of the clinical spaces that had been previously assigned for in-situ simulation training.

During the first training session, proctors completed 25 checklists and 292 out of 391 (75%) critical action checklist items were completed by the teams. The Appendix presents the checklists used for the study. Noted gaps in the critical action checklist for the obstetrics care component included quickly identifying that the standardized patient had a twin pregnancy, communication between obstetrics and pediatrics team members with the transport of standardized patient to the inpatient unit, and consideration of packing the abdomen before proceeding with hysterectomy to allow the anesthesia team to improve the simulated patient’s fluid and blood product resuscitation. Role delineation and task saturation were frequent concerns for nursing. Thermoregulation and the appropriate placement of the neonate for resuscitation and the activation of emergency release blood were common gaps identified from the newborn resuscitation scenarios.

A lack of non-technical skills of the anesthesia participants was highlighted during the simulated scenarios. Anesthesia participants consisted mostly of anesthesia trainees, both anesthesiology residents and student registered nurse anesthetists. Some trainees participated by themselves and others participated with a credentialed staff member but were required to play the role of the lead provider. Once the anesthesia trainee understood the clinical situation and the necessity of the simulated parturient to go to the operating room for an emergent cesarean section, they were able to focus their actions to prepare the patient for surgery. Once inside the operating room, the trainees were given three minutes to place a spinal anesthetic in a partial task trainer. If they were unable to do so in the allotted time, the proctor stated that the fetus was decompensating and a general anesthetic must be administered. The anesthesia trainee then had to perform a rapid sequence induction and place the endotracheal tube. The most commonly missed critical steps were categorized as the non-technical skills of the anesthesia trainee. Many participants worked independently without much communication with the nursing or obstetric team. Oftentimes, when the trainee recognized he or she needed more information or help, they lacked the assertiveness to get the attention they needed. It was quickly realized by many of the anesthesia participants that other team members did not know how to provide the help or resources they needed. Help with placing additional intravenous lines, initiating the infusion of blood products, or requests to get equipment such as the rapid fluid infuser or the “hotline” was met with staff that did not know where to find the equipment or didn’t know the combination to get access to secured spaces where the equipment was kept.

Through the structured debrief, the evaluation of unmet critical action items, and proctor observation, participants and proctors identified 34 latent safety threats, which were categorized as training needs, process needs, equipment needs, and visual needs. The specifics of these “needs” are presented in Tables 1-2. The four categories of needs continued to be addressed within the hospital system and perinatal care units during this first training session. The 34 latent safety threats were prioritized and plans of action were developed to mitigate these threats. The staff was educated and corrective actions implemented. An example of the corrective action involved relocating the Belmont® Rapid Infuser (Belmont Medical Technologies, MA, US), which was moved from a locked anesthesia storage room to the primary labor and delivery operating room. This immediately improved access and decreased the delay of care.

**Table 1:** Specific Latent Safety Threats Identified Through In-Situ Perinatal Safety Simulation TeamSTEPPS: Team Strategies and Tools to Enhance Performance and Patient Safety

Training Needs	Process Needs
Portable perinatal emergency bag contents	Inventory/stocking/locking of perinatal emergency bag
Handheld radio operations	Security: Crowd control (Prevent inadvertent infant security code and secure elevators for transport)
Belmont and Ranger operations	Massive transfusion protocol process for unadmitted patient/computer downtime
Local anesthesia protocol	Unadmitted patient process
Massive transfusion protocol vs emergency release blood protocol	Emergency Department response to perinatal emergency
Use of closest elevator for transport	Identification/color coding of team members
Communication issues/TeamSTEPPS tools	Baby bands added to perinatal emergency bag inventory
Use of Doppler to reassess fetal heart tones in transit	Ensure third Labor and Delivery operating room routinely stocked for emergencies
Blood release process (2 requests=2 runners)	
Speed dial for operating room overhead paging	
Glidescope location	
Orientation to operating rooms	

**Table 2:** Specific Latent Safety Threats Identified Through In-Situ Perinatal Safety Simulation (cont.)

Equipment Needs	Visual Needs
Operating room wall clocks	Post operating room phone numbers at front desk
Markers for operating room boards	Blood bank/Neonatal Intensive Care Unit/front desk numbers posted in operating rooms
2 way radios with dedicated labor and delivery channel/chargers	“Poor man’s coagulation” reference in operating rooms
Caps/masks at operating room entrance	Local anesthesia protocol
Laryngoscope blades/handles	
Backboard	
Transport gurney	
Oxygen tank	
Consider portable monitor	

The second training session involved four teams (45 staff members). Only 15 of these staff members had participated in the initial training session. Proctors completed six checklists and 94 out of 106 (89%) critical action checklist items were completed, for an overall checklist compliance of 89% for the second training session. Ten of the previously identified latent safety threats from the first training session were mitigated during this second training session (Table 3). Utilizing a z-ratio, a significant difference was detected between the overall checklist compliance rates between the two training sessions, z = -3.069, p = .002. However, a post hoc power analysis indicated the study lacked power (<10%), as 84 checklists would have been needed in each group to detect a 14% improvement with 80% power. Of the 105 participants, 88 (84% response rate) completed post-training surveys and self-reported that the simulation training emphasized the importance of communication and teamwork. The primary learning points from the in-situ simulation included reinforcement of TeamSTEPPs' principles involving team communication. On a Likert scale with anchors (1=strongly disagree to 5=strongly agree), participants agreed or strongly agreed with the statement that the simulation training provided a team-building experience (means 4.6 and 4.44, training sessions one and two, respectively) and would improve patient safety (means 4.67 and 4.33, training sessions 1 and 2, respectively). Results are presented in Table 3. There was no significant difference in the mean scores between the training sessions but a majority of the participants participated in only one of the two training sessions.

**Table 3:** Impact of In-Situ Perinatal Simulation Training *Only 15 learners participated in both training events †z-ratio ‡Likert scale anchors (1=strongly disagree, 5=strongly agree); Mann-Whitney Test

Measure	Initial Training	Subsequent Training	P
Staff Participants (Teams)	75 (7)	45* (4)	
Checklists (% Checklist Compliance†)	25 (75%)	6 (89%)	0.002†
Latent Safety Threats Identified	34	24	
Participant Experience: Team Building, mean (median)	4.60 (5)	4.44 (4)	0.362‡
Participant Experience: Improve patient safety, mean (median)	4.67 (5)	4.33 (4)	0.071‡

Patient satisfaction surveys completed six months before the initial training were compared to the surveys completed six months after the initial training to evaluate patient perceptions of their care. Patient perceptions of their care improved by more than 5% for all assessed categories. Results are presented in Table 4. The question most germane to this study addressed the “competency of clinical staff in performing their jobs” or simply staff competency. Patient perceptions of staff competency improved from 4.6 to 4.86, an increase of 5.65%. To determine significance, a two-sample (unpaired) t-test was used. However, due to constraints with the survey data, a liberal standard deviation needed to be estimated (SD=1.1) for the calculation. Results from the t-test were significant (p =0.048).

**Table 4:** Patient Perception Ratings Before and After In-Situ Perinatal Simulation Training ‡Likert scale anchors (1=strongly disagree, 5=strongly agree)

Measure	Six Months Before Training	Six Months After Training	% Change
Employee/Staff Attitude, Mean Rating‡ (#responses)	4.59 (86)	4.84 (424)	+5.4%
Caring Manner of Clinical Staff, Mean Rating‡ (#responses)	4.55 (86)	4.84 (424)	+6.4%
Staff competency, Mean Rating‡ (#responses)	4.6 (84)	4.86 (477)	+5.7%

## Discussion

This study demonstrated the utility of structured debriefs as evident by the in-situ perinatal multi-disciplinary perinatal team identifying 34 latent safety threats that could potentially impact the quality of care a patient would receive across the continuum of care. The structured debrief also allowed for the development of action plans but, most importantly, the structured debrief facilitated the mitigation of latent safety threats and improvement in emergency checklist compliance with repeated training. These findings are also consistent with previous research, which has shown that the implementation of a surgical safety checklist is associated with improvements in communication, leadership, and the ability to be assertive when necessary to improve patient safety [24].

In order to improve patient outcomes in a health system, one must look at not only the competency and performance of the team providing direct medical care but also the processes within a health system across the continuum of care [25]. Current techniques used to evaluate processes within a health system include leadership rounds and patient tracers [26-27]. While leadership rounds can evaluate operating procedures and the knowledge of the team prior to the provision of care, it remains difficult to evaluate the team’s implementation of procedures and system processes before a patient actually interacts with the health system.

In-situ simulation training brings the simulation scenarios and drills into the clinical workspace, to improve the fidelity of the environment, system, and processes in which the health care team functions [13]. In addition to allowing the team to develop new skills or maintain infrequently used skills, a more recently reported trend uses in-situ simulation scenarios to identify latent safety threats within a unit or a health system [11-15]. These latent safety threats function as a type of lead measure on which unit and health system leadership can initiate change proactively rather than waiting for the evaluation of patient safety events to implement changes (a type of lag measure). Acting on lead measures not only addresses patient safety threats before adverse events occur but also allows increased momentum as a healthcare system becomes a high-reliability organization [28].

Challenges do exist with performing in-situ training on a busy clinical unit; however, having a smaller, separate training team from the team assigned to clinical care on the unit for the day, maintaining good communication between the training team and the team providing clinical care on the unit, and a flexible simulation team can offset those challenges and allow the successful implementation of the training. One benefit of interprofessional team training is that multiple components of team performance can be assessed during the training. While this project was significantly underpowered, we were able to gain some valuable information. Through the use of specialty-specific and interprofessional emergency checklists to assess each team, one may increase the rigor and use the number of checklists as a potential tool on which to power future studies rather than only on the number of teams that are trained, a potential limiting step at some institutions.

In-situ simulation training allows for the proactive, systematic identification of latent safety threats that can be addressed before those threats become patient safety “near misses” or sentinel events or are identified through a root cause analysis. Furthermore, in-situ training allows for the implementation of skills gained through rehearsal using task trainers and multidisciplinary team training based in a simulation center in the actual clinical environment, increasing the fidelity of the training.

The strengths of our study include the use of standardized simulation mannequins, scenarios, and emergency checklists during the in-situ training sessions. Institutional surveys were used to evaluate participant and patient experiences to determine the impact of in-situ simulation training. Formal structured debriefs were developed and implemented to identify knowledge gaps with checklist compliance and patient safety.

Our study has certain limitations. First, the design of this study resulted in a small number of groups, which was adequate to detect checklist compliance performance but had limited statistical power (post hoc power analysis < 10%). Thus, a true difference between checklist performances between the two sessions was most likely under-detected (type II error). Second, the authors attempted to maintain scenario consistency between the two sessions by utilizing the same scenarios in both sessions. However, it is possible that the two scenarios were not entirely equal. This could have resulted in unequal checklist performance between the two groups. Third, the use of non-validated surveys for participants and patients prevent generalizability. Fourth, participant samples within each group were small and multiple surveys had to be discarded due to missing responses. Furthermore, the lack of a significant improvement in patient satisfaction is also likely due to low power. Additional limitations of our study include the sample size with a lack of paired data to determine a true pre-/post-effect in the evaluation of the participant experience.

## Conclusions

The implementation of a structured debrief during in-situ perinatal interprofessional simulation training was associated with a statistically significant increase with emergency checklist compliance. Structured debriefing allowed for the identification and mitigation of latent patient safety threats during the simulation training. Results also indicate that in-situ perinatal safety training contributes to improved patient perception of staff clinical competency and staff rating of patient safety. Other medical centers may desire to develop interprofessional in-situ simulation scenarios to identify latent threats specific to their units and environment of care. They could then test process improvements within a health system as they move toward becoming high-reliability organizations with an emphasis on healthcare improvement. Further research should evaluate how to effectively implement structured debriefing into other training scenarios to evaluate improvements in teamwork and communication among training teams that stems from emergency checklist compliance, which could ultimately result in reductions in postoperative morbidity and mortality.

## Appendices

**Table 5:** Obstetrics Critical Action Checklist

Obstetrics Critical Action Checklist (I/II)	Met	Not Met	Comments
Code Purple team arrives & asks patient gestational age, pregnancy complications for imminent delivery			
Delivers preterm infant			
Hand-off ( Situation, Background, Assessment, Recommendation) to peds team			
Checks to see if other twin delivery is imminent/presentation			
Transfers to Labor and Delivery/Operating room			
Places Twin B on monitor			
Ultrasound to document fetal presentation			
Communicates need for cesarean delivery but reassuring status for spinal attempt			
3 minute terminal fetal bradycardia; Obstetric team communicates need to proceed with cesarean emergently under general anesthesia if spinal not yet obtained.			
Cesarean delivery via low vertical/classical uterine incision– communicates to anesthesia and peds likely abruption			
Poor uterine tone noted; begins postpartum hemorrhage algorithm; requests activation of massive transfusion protocol			
12) Uterotonics - Medications (double pitocin, hemabate, methergine, Tranexamic acid)			
13) Nonsurgical options discussed - Bakri balloon			
14) Surgical options discussed/implemented (bilateral O'Leary, B-lynch, hypogastric artery ligation, prep for cesarean hysterectomy)			
15) consideration of packing abdomen to wait for anesthesia to improve resuscitation.			

**Table 6:** Obstetrics Critical Action Checklist (Cont)

Obstetrics Critical Action Checklist (II/II)	Met	Not Met	Comments
Begins chest compressions when notified by anesthesia of code? Calls code blue if not already done…becomes part of ACLS team for resuscitation.			
Recognizes Ventricular Fibrillation			
High-quality cardiopulmonary resuscitation			
Clears before analyze and shock			
Immediately resumes chest compressions aftershocks			
Airway management			
Appropriate cycles of drug-rhythm check/shock-chest compressions			
Administers appropriate drug(s) and doses			
Recognizes pulseless electrical activity			
Verbalizes potential reversible causes of pulseless electrical activity			
Administers appropriate drug(s) and doses			
Immediately resumes chest compressions after rhythm and pulse checks			
Identifies return of spontaneous circulation			
Ensures blood pressure/12-lead electrocardiogram is performed, oxygenation monitored, orders labs tests			
Targeted temperature management			
intensive care unit bed			

**Table 7:** Neonatal Critical Action Checklist

Multi-Disciplinary Neonatal Critical Action Checklist			
Twin A: Pre-term Vaginal Delivery Outside of Labor and Delivery	Objective Met	Objective Not Met	Comments
Initial assessment after delivery. Infant depressed with no respiratory effort.			
Address thermoregulation and placement of infant for resuscitation.			
Follow Neonatal Resuscitation Protocol for initial stabilization			
Identify pneumothorax and treat accordingly			
Apply proper use of S.T.A.B.L.E. Program			
Twin B: Pre-term C-section in the Operating Room	Objective Met	Objective Not Met	Comments
Initial assessment after delivery. Infant depressed with no respiratory effort.			
Address thermoregulation and placement of infant for resuscitation.			
Follow Neonatal Resuscitation Protocol for initial stabilization			

**Table 8:** Nursing Critical Action Checklist

Obstetric Emergency Simulation Nursing Evaluation			
	Met	Not Met	Comments
1. Assesses the patient			
2. Assists Provider/patient as needed a. Delivery b. locates cord clamps, scissors, blankets/towels			
3. Obtain Vital signs			
4. Obtains intravenous access			
5. Ensures collection of blood bank specimen and its immediate transport to the blood bank			
6. Explain all procedures and plans to patient			
7. Effectively communicates to labor and delivery charge nurse: a. patient info b. transport to labor and delivery operating room c. immediate needs (ultrasound machine, extra personnel, delivery table, surgical tech, etc..)			
8. Pre-surgical needs: a. fetal monitoring/ultrasound b. right hip roll c. antibiotics d. foley e. bovie pad f. sequential compression devices g. surgical prep			
9. Abbreviated Time out a. patient name/date of birth b. procedure c. allergies			
10. SBAR to neonatal team			
11. Proper initiation of the massive transfusion protocol per Physician order			
12. Assists Anesthesia with additional intravenous line/Cricoid Pressure			
13. Demonstrates proper pad placement and operation of defibrillator			
14. Ensures cancellation of the massive transfusion protocol as directed			

**Table 9:** Multidisciplinary Evaluation

Obstetric Emergency Simulation Multidisciplinary Evaluation			
Team Performance	Met	Not Met	Comments
Shared Mental Model			
1. All SBAR elements present			
2. Did team “receive” the report?			
3. Did all team members get report as they arrived?			
Role Clarity			
1. Clear Nursing and Physician Leader			
2. Roles clearly established through self-assignment or team leader assigning roles?			
3. Are roles clear to ALL team members			
Situational Awareness			
1. Ongoing monitoring and crosschecking (assesses condition and response to treatment)			
2. Recognizes critical information in a timely fashion			
3. Effective assertion to appropriate team member? (2 challenge rule if needed?)			
Closed Loop Communication			
1. Leader responds to input from the team			
2. Read back of all orders			
Psychomotor Skills/Equipment Competency			
1. Quickly locates critical equipment			
2. Competent with equipment and supplies			

**Table 10:** Anesthesia Critical Action Checklist

Obstetric Emergency Simulation Anesthesia Evaluation			
Critical Steps for General Anesthesia and BLOOD TRANSFUSION IN EMERGENT CESAREAN DELIVERY (I/II)	Objective Met	Objective Not Met	Comments
1. Communicates with surgeon to determine urgency of procedure			
2. Assesses the patient to obtain pertinent medical and obstetric history i.e. allergies medications complications during pregnancy, vital signs surgical hx. Airway exam family anesthesia hx. Etc.			
3. IF not previously done; checks equipment and monitors function/availability			
4. Preoperative patient care to include left uterine displacement, sodium citrate, 100% FiO2, patent intravenous catheter, vital signs			
5. Induction: Verify obstetric team readiness Apply cricoid pressure Administer induction agent and succinylcholine Direct /Video laryngoscopy Pass endotracheal tube/Inflate cuff Confirm presence of end-tidal CO2 Notify surgeon to proceed Confirm bilateral breath sounds Release cricoid pressure Secure endotracheal tube			
6. Before Delivery: Initiate mechanical ventilation Appropriate tidal volume/respiratory rate Maintain FiO2>0.5 Maintain inhaled agent > 1 MAC Protect eyes Place and suction orogastric tube Monitor temperature Assess neuromuscular blockade			

**Table 11:** Anesthesia Critical Action Checklist (Cont)

Obstetric Emergency Simulation Anesthesia Evaluation			
Critical Steps for General Anesthesia and BLOOD TRANSFUSION IN EMERGENT CESAREAN DELIVERY (II/II)	Objective Met	Objective Not Met	Comments
7. After Delivery: Initiate infusion of oxytocin Decrease inhaled agent to < 0.5 MAC Administer nitrous oxide, opioids, and muscle relaxants as needed			
8. Continually assesses patient’s hemodynamic status and blood loss. Maintains constant communication with surgeon in regards to hemostasis			
9. Uncontrollable bleeding: Establishes two large bore intravenous catheters if not placed already Volume resuscitation with warmed crystalloid and/or colloid Determine need for immediate transfusion Calculate total requirements Determined if crossmatched packed red blood cells or massive transfusion protocol necessary Request crossmatched packed red blood cells or massive transfusion protocol (6 units packed red blood cells, 4 units Fresh Frozen Plasma, 1 unit platelets) Obtain labs: Hemoglobin/Hematocrit/platelets, Prothrombin/Partial Thromboplastin, Fibrinogen, D Dimer, international normalized ratioAdminister blood products Assess ongoing bleeding Deactivate massive transfusion protocol if initiated when hemostasis occurs Repeat labs			
